# Observation and quantitative analysis of dislocations in steel using electron channeling contrast imaging method with precise control of electron beam incident direction

**DOI:** 10.1093/jmicro/dfad061

**Published:** 2023-12-19

**Authors:** Takashige Mori, Takafumi Amino, Chie Yokoyama, Shunsuke Taniguchi, Takayuki Yonezawa, Akira Taniyama

**Affiliations:** Research & Development, Nippon Steel Corporation, 1-8 Fuso-Cho, Amagasaki, Hyogo 660-0891, Japan; Research & Development, Nippon Steel Corporation, 1-8 Fuso-Cho, Amagasaki, Hyogo 660-0891, Japan; Research & Development, Nippon Steel Corporation, 1-8 Fuso-Cho, Amagasaki, Hyogo 660-0891, Japan; Research & Development, Nippon Steel Corporation, 1-8 Fuso-Cho, Amagasaki, Hyogo 660-0891, Japan; Research & Development, Nippon Steel Corporation, 20-1 Shintomi, Futtsu, Chiba 293-8511, Japan; Research & Development, Nippon Steel Corporation, 1-8 Fuso-Cho, Amagasaki, Hyogo 660-0891, Japan

**Keywords:** SEM, ECCI, dislocation, steel, TEM, EBSD

## Abstract

Electron channeling contrast imaging (ECCI) was applied by precisely controlling the primary electron beam incident direction of the crystal plane in scanning electron microscope (SEM), and the dislocation contrast in steel materials was investigated in detail via SEM/ECCI. The dislocation contrast was observed near a channeling condition, where the incident electron beam direction of the crystal plane varied, and the backscattered electron intensity reached a local minimum. Comparing the dislocation contrasts in the visualized electron channeling contrast (ECC) images and transmission electron microscope (TEM) images, the positions of all dislocation lines were coincident. During the SEM/ECCI observation, the dislocation contrast varied depending on the incident electron beam direction of the crystal plane and accelerating voltages, and optimal conditions existed. When the diffraction condition **g** and the Burgers vector **b** of dislocation satisfied the condition **g⸱b **= 0, the screw dislocation contrast in the ECC image disappeared. An edge dislocation line was wider than a screw dislocation line. Thus, the SEM/ECCI method can be used for dislocation characterization and the strain field evaluation around dislocation, like the TEM method. The depth information of SEM/ECCI, where the channeling condition is strictly satisfied, can be obtained from dislocation contrast deeper than 5*ξ*_g_, typically used for depth of SEM/ECCI.

## Introduction

Electron channeling in scanning electron microscopy (SEM) is directly related to crystallographic orientation relative to the incident electron beam. Moreover, the electron channeling SEM images can provide information on the crystallographic properties of a sample [1,2]. Coates [3] first reported that Kikuchi-like reflection patterns were observed in structural images of single-crystal samples obtained by backscattered electrons (BSE) at relatively low magnifications. Subsequently, several qualitative understandings were given by Booker [4] and Hirsch [5], and theoretical interpretations have been discussed so far [6–10]. If electron channeling can observe the local crystal orientation changes, capturing the changes in the BSE intensity should be possible due to lattice defects such as grain boundaries and dislocations in a well-polished bulk sample [5,11]. Several examples are reported regarding the observation of lattice defects in bulk samples [12–23] using electron channeling contrast imaging (ECCI).

Furthermore, attempts were made to identify the crystal defect contrast in polycrystalline materials observed by SEM/ECCI by comparing with transmission electron microscopy (TEM); Zauter *et al*. [24] observed the dislocation structure formed in austenitic stainless steels after fatigue testing by SEM/ECCI and TEM and identified similar characteristic subgrains. Weidner *et al*. [25] and Sugiyama *et al*. [26] observed the steel after deformation by SEM/ECCI and TEM, reporting similar characteristics of crystalline defects. Zaefferer *et al*. [10] directly compared bright-field (BF) TEM and ECC images obtained from the same region of the TEM foil of twining-induced plasticity steel and showed the observation results for the contrast corresponding to dislocation, stacking fault, ε-martensite lamella, and a large slope of stacking fault; distinguishing the dislocation contrast from the surface roughness contrast was difficult. Pang *et al*. [27] compared SEM/ECCI and TEM dislocation contrasts in the same region of a TEM sample of impacted tantalum. They showed that all the dislocation contrasts observed in SEM/ECCI were also observed in TEM; however, many dislocation lines in the TEM image were absent in the ECC image. Thus, although there are many examples of direct comparison of crystal defect contrasts between SEM/ECCI and TEM, few show a clear correspondence between their dislocation contrast.

The orientation relationship between the incident electron beam and crystal must be precisely controlled up to the channeling condition where the rocking curve of the backscattered electron intensity reaches a local minimum [10] to observe dislocation contrast in any region using SEM/ECCI. Mansour *et al*. [28,29] proposed accurate ECCI (A-ECCI), which accurately measures crystal orientation information in regions satisfying channeling conditions. This method is an effective means of knowing the correct diffraction conditions during observation using selected area channeling patterns. However, controlling the desired diffraction conditions is difficult. L’hȏte *et al*. [30–32] proposed rotational ECCI (R-ECCI), recording a series of images while rotating the sample to obtain the BSE intensity profile. However, accurate diffraction conditions could not be obtained for low-magnification scans because the electron beam incident direction shifted depending on the beam position. Zaefferer *et al*. [10,33] proposed ECCI under controlled diffraction conditions (cECCI), calculating the tilt and rotation angles of the SEM sample stage required to achieve the desired channeling conditions based on the crystal orientation information previously obtained by electron backscatter diffraction (EBSD); this method helps determine the optimal stage tilt angle for achieving the channeling condition. However, it is not known whether the set diffraction conditions are correctly excited because of the SEM stage uncertainty. In addition, when direction control is used, the observation area moves with stage rotation, causing the image to rotate; these corrections are necessary.

Nevertheless, few studies have examined the effects of the electron beam incidence direction on the dislocation contrast in SEM/ECCI. Pang *et al*. [27] simulated the contrast of a screw dislocation in tantalum as a function of deviation parameter *w*. Kriaa *et al*. [34] observed the difference in the dislocation width due to changes in the incident orientation of primary electron beams along the Kikuchi band with the same diffraction vector **g**. Gutierrez [35] experimentally investigated the influence of *w* on the dislocation contrasts, concluding that the channeling conditions with large positive values of *w* (*w *> 1.5 ± 0.5) are optimal for imaging dislocation contrast in SEM/ECCI.

In this study, SEM/ECCI observation was conducted by controlling the incident electron beam direction with high precision using a piezoelectric element as a driving unit; the dislocation contrast in steel materials in SEM/ECCI was investigated, and the depth information of the dislocation contrast in SEM/ECCI was measured experimentally.

## Experimental methods

Fe–0.13C–0.26Si–1.3Mn–0.014P–0.003S (mass%) steel sample was used in this study. By heating the steel ingot to 1100℃, hot rolling at a finishing temperature of 750℃, and cooling to room temperature, the ferrite/pearlite microstructure was obtained. The surface of the steel sample was mirror-polished by mechanical polishing with emery paper and buffing with diamond slurry and colloidal silica. The crystal orientation of the ferrite grains on the sample surface was determined by EBSD. Then, a Kikuchi map, a simulated electron channeling pattern of the observation area, was calculated from the Euler angles taken by EBSD and the crystal structures representing the crystal orientation of the analyzed points. The channeling conditions suitable for observing dislocations by SEM/ECCI were satisfied near the Bragg conditions [10]. Therefore, simulated pseudo-Kikuchi lines constituting the Kikuchi map were drawn with a width twice than the Bragg angle *θ*_B_. The intensity of the simulated pseudo-Kikuchi line is the square of the crystal structure factor *F,* obtained by assuming that the diffraction intensity of the crystal lattice, follows the dynamical theory and is drawn to be proportional to the brightness of the simulated pseudo-Kikuchi line on each surface. For simplicity, only simulated pseudo-Kikuchi lines with intensities up to the 10^th^ rank were drawn in this study, and the second- and higher-order reflections were not drawn.

SEM/ECCI was performed based on the Kikuchi map by tilting the sample in a JEOL JSM-7800 F SEM using a special tilting stage with three piezoelectric axes to precisely control the incident electron beam direction to the crystal plane. ECC images were obtained using a standard bottom-column BSE detector with 4 mm working distance, 30 µm aperture and 15–30 kV accelerating voltages; EBSD measurements were performed with an EDAX/TSL system with a Hikari camera and TSL Data collection software version 7.01. at 25 kV accelerating voltage. In addition, a focused ion beam (FIB) thin-film sample prepared using a FEI Helios NanoLab 600i consisting of a FIB gun and field-emission SEM was observed by a FEI Tecnai F30 TEM at 300 kV. Then, SEM/ECCI was applied to the same area of the TEM-observed thin-film samples.

## Results and discussion

### Influence of the angle of the primary electron beam direction in SEM/ECCI

**Fig. 1.**
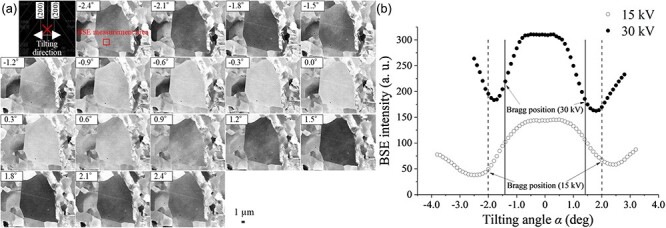
BSE intensity as a function of the primary electron beam direction to (200) crystal plane of a ferrite grain; (a) electron channeling contrast images of ferrite grain obtained by changing the incident electron beam direction to (200) crystal plane of observed grain at 30 kV accelerating voltage; (b) BSE intensity versus tilting angle *α* of the sample around tilting axis parallel to the (200) crystal plane at 15 and 30 kV.

Figure 1(a) shows ECC images of ferrite grains obtained by changing the incident primary electron beam direction to the (200) crystal plane of the observed grain, at an accelerating voltage of 30 kV, and the BSE intensity versus tilting angle *α* of the sample around the tilting axis parallel to (200) crystal plane at 15 and 30 kV. The BSE intensity was measured using the average of 100 × 100 pixels in the same area of each image (square area, Fig. 1(a)). As the incident primary electron beam direction angle varied concerning the (200) crystal plane, the BSE intensity changed significantly near the Bragg conditions. In the present investigation, the width of the tilting angle between the Bragg positions of (200) and $(\overline 2 00)$ of ferrite was ∼2.8° at 30 kV. However, the width of the tilting angle between the channeling positions of (200) and $(\overline 2 00)$ was ∼3.6°, the incident condition where the BSE intensity reached a local minimum. These experimental results were in good agreement with the calculated rocking curves of the backscattered diffraction intensities near the Bragg conditions [10,11,27], where the channeling conditions were satisfied with a deviation parameter *w *> 0, i.e. on the higher-angle side rather than the Bragg condition. Even at 15 kV, the channeling condition was satisfied at the higher-angle side rather than 30 kV as the Bragg angle changed.

**Fig. 2.**
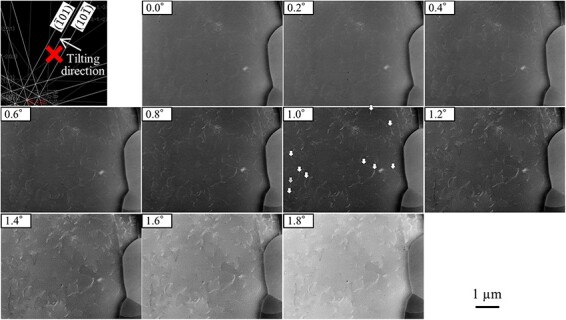
Electron channeling contrast images obtained by changing the incident electron beam direction to $(10\overline 1 )$ crystal plane of ferrite grain at 30 kV.

**Fig. 3.**
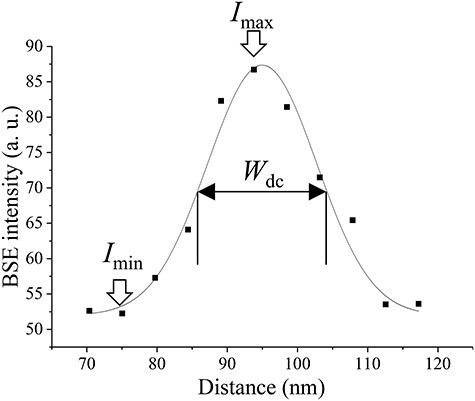
Evaluation of dislocation contrast intensity $\textstyle{\mathrm I}_{\mathrm d\mathrm c}$ and width $\textstyle{\mathrm W}_{\mathrm d\mathrm c}$; BSE intensity profile in a 100 nm long region of interest (ROI) set along a dislocation: the width of ROI depends on each dislocation (Fig. 2, gray arrow); $\textstyle{\mathrm I}_{\mathrm d\mathrm c}$ = ($\textstyle{\mathrm I}_{\mathrm m\mathrm a\mathrm x}$-$\textstyle{\mathrm I}_{\mathrm m\mathrm i\mathrm n}$)/($\textstyle{\mathrm I}_{\mathrm m\mathrm a\mathrm x}$+$\textstyle{\mathrm I}_{\mathrm m\mathrm i\mathrm n}$), where $I_{\mathrm m\mathrm a\mathrm x}$ and $\textstyle{\mathrm I}_{\mathrm m\mathrm i\mathrm n}$ are the highest and lowest values of BSE intensity profile across a dislocation; $\textstyle{\mathrm W}_{\mathrm d\mathrm c}$ is evaluated as the full width at half maximum obtained with the Gaussian fit of the line profile.


**Fig. 4.**
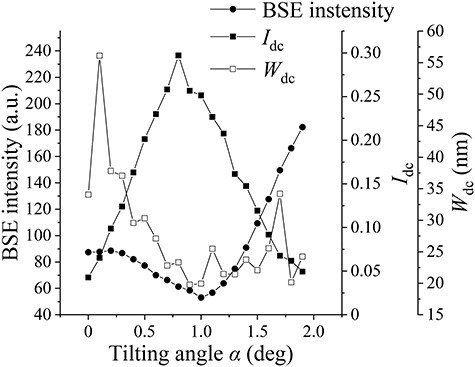
BSE intensity and dislocation contrast as a function of tilt angle *α* around the Bragg position of $\textstyle\mathbf g=10\overline1$; BSE intensity, dislocation contrast intensity $\textstyle{\mathrm I}_{\mathrm d\mathrm c}$, and dislocation contrast width $\textstyle{\mathrm W}_{\mathrm d\mathrm c}$ vs. sample tilt angle *α* around tilt axis parallel to $(10\overline 1 )$ crystal plane at 30 kV.

The dislocation observation by SEM/ECCI requires channeling conditions where the BSE intensity takes a local minimum. Therefore, the dislocation contrast near the channeling condition was investigated. Figure 2 shows ECC images obtained by changing the incident electron beam direction to $(10\overline 1 )$ crystal plane of the ferrite grain at 30 kV. The dislocation contrast was varied by changing the tilting angle *α* near the channeling condition. The dislocation contrast intensity $\textstyle{\mathrm I}_{\mathrm d\mathrm c}$ and width $\textstyle W_{\mathrm d\mathrm c}$ were evaluated to quantitatively investigate the dislocation contrast near the channeling condition. Figure 3 shows an example of the BSE intensity profile across the dislocation shown in Fig. 2 by the gray arrow. A line profile across a dislocation was obtained by calculating the mean of the BSE intensity profile in a 100 nm long region of interest (ROI) set along a dislocation. The width of ROI depends on each dislocation. $\textstyle{\mathrm I}_{\mathrm d\mathrm c}$ was estimated following Equation 1 [35]:

(1) $$I_{\mathrm d\mathrm c}=\frac{I_{\mathrm m\mathrm a\mathrm x}-I_{\mathrm m\mathrm i\mathrm n}}{I_{\mathrm m\mathrm a\mathrm x}+I_{\mathrm m\mathrm i\mathrm n}}$$

where $I_{\mathrm m\mathrm a\mathrm x}$ and $I_{\mathrm m\mathrm i\mathrm n}$ are the highest and lowest values of the BSE intensity profile across a dislocation, respectively. In this study, $W_{\mathrm d\mathrm c}$ was evaluated as the full width at half maximum (FWHM) obtained from the Gaussian fit of the line profiles of dislocations. $I_{\mathrm d\mathrm c}$ and $W_{\mathrm d\mathrm c}$ were estimated regarding the 10 dislocations shown in Fig. 2 by the opened arrows from the series of ECC images obtained by changing the primary electron beam direction to the $(10\overline 1 )$ crystal plane of the ferrite grain at 30 kV. Then, $I_{\mathrm d\mathrm c}$ and $W_{\mathrm d\mathrm c}$ of the same ECC image of the tilting angle were calculating the means of them and plotted in Fig. 4 with BSE intensity as a function of the tilting angle *α*. High $I_{\mathrm d\mathrm c}$ and low $W_{\mathrm d\mathrm c}$ are good values for observing dislocations in SEM/ECCI. $I_{\mathrm d\mathrm c}$ and $W_{\mathrm d\mathrm c}$ varied by changing the tilting angle *α*. $W_{\mathrm d\mathrm c}$ tended to have a peak bottom near the channeling condition. However, a peak top of $I_{\mathrm d\mathrm c}$ was shifted from the channeling condition where the BSE intensity reached a local minimum. This experiment result showed that the strongest dislocation contrast could be obtained at higher *w* than the channeling condition. In the channeling condition, difference in the dislocation line width depending on dislocations of interest existed, potentially to correspond to the difference in the strain field due to dislocation characters.

**Fig. 5.**
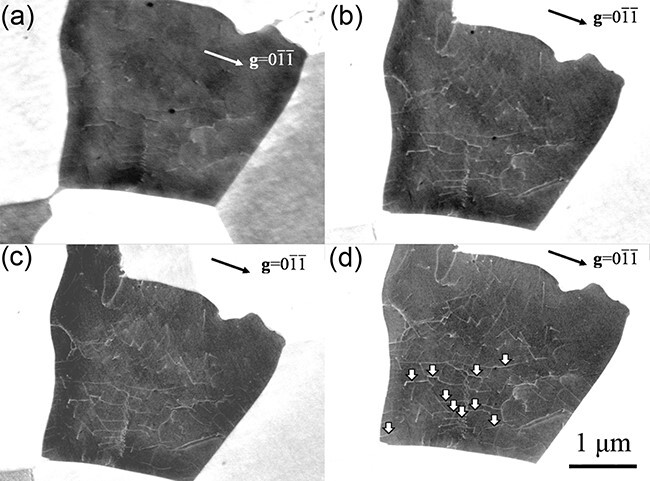
ECC images of the same field of view obtained under stepwise accelerating voltages of (a) 15, (b) 20, (c) 25 and (d) 30 kV, strictly satisfying channeling conditions with ${\bf{g}} = 0\overline 1 \overline 1 $ excitation.

**Fig. 6.**
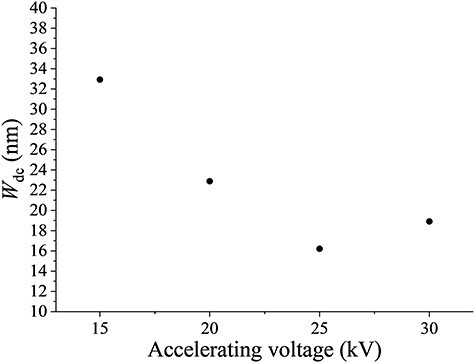
Dislocation contrast width $W_{\mathrm d\mathrm c}$ as a function of accelerating voltages.

Few studies on the effects of the accelerating voltage on the dislocation contrast in SEM/ECCI [33,36]. Moreover, whether the contributions of high accelerating voltages are positive or negative is still under discussion. Therefore, observing the dislocation contrast by SEM/ECCI has been operated at accelerating voltage of 10–30 kV [15–23], but the optimal conditions of accelerating voltages are still unclear. As mentioned above, the channeling position varies with the accelerating voltage value. Therefore, repeating the directional control to the channeling conditions when comparing the same field of view is necessary. Figure 5 shows ECC images of the same field of view obtained under a stepwise accelerating voltage of 15–30 kV and strictly satisfied channeling conditions. $W_{\mathrm d\mathrm c}$ was evaluated like the method described above using 10 dislocations (Fig. 5, opened arrows) in ECC images of 15–30 kV, and averaged $W_{\mathrm d\mathrm c}$ on accelerating voltage was plotted in Fig. 6. Note that $I_{\mathrm d\mathrm c}$ cannot be used in this case, due to the brightness and contrast of ECC images that were indispensably adjusted since the BSE intensity varied with accelerating voltages. From 15 to 25 kV, $W_{\mathrm d\mathrm c}$ decreased as the accelerating voltage increased. As for 25–30 kV, $\textstyle W_{\mathrm d\mathrm c}$ increased at 30 kV. The higher the applied accelerating voltage, the more abruptly the BSE intensity tilted around the Bragg position changes (Fig. 1). Therefore, in this case, the change in the BSE intensity associated with local crystal rotation around the dislocation line in the channeling state was more abrupt at high acceleration voltages until 25 kV. Furthermore, the probe size of the electron beam was considered a factor affecting $W_{\mathrm d\mathrm c}$. In this study, all ECC images were taken under the same WD and aperture size, and the convergence semiangle of the electron probe was the same. However, the convergence semiangle of the electron probe minimizing the probe size, differed depending on accelerating voltages, mainly due to the influence of diffraction aberration and spherical aberration at high accelerating voltages [2]. Therefore, 30 kV $W_{\mathrm d\mathrm c}$ was considered worse than 25 kV. Since the convergence semiangle of the electron probe varied depending on aperture size, WD, and the electron optical system of devices, setting the optimal observation conditions along with controlling the incident electron beam direction is necessary.

### Direct comparison of dislocation contrast between SEM/ECCI and bright-field TEM

**Fig. 7.**
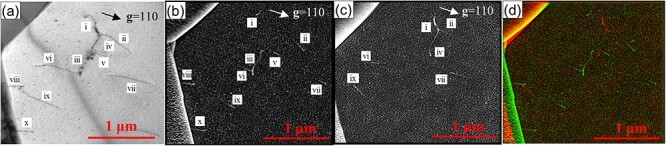
Direct comparison of details in the dislocation contrast of bright-field (BF) TEM and ECC images; (a) BF-TEM image with **g **= 110 excitation from front side of the thin film at 300 kV, (b) ECC image with **g **= 110 from front side at 30 kV, (c) left and right reversed ECC images with **g **= 110 from back side at 30 kV and (d) a superimposed image of (b) and (c).

BF-TEM observations were performed on the same thin film of a ferrite grain to validate SEM/ECCI as a method for observing dislocation contrast. Figure 7(a) shows the BF-TEM image of a ferrite grain obtained with **g **= 110 excitation from the front side of the thin film. Figures 7(b)–(d) show ECC images of the ferrite grain obtained with exciting **g **= 110 from the front and back sides of the thin film. Each dislocation with endpoints was observed independently on the front and back sides of the thin film in BF-TEM images. Herein, the dislocations in the BF-TEM image were numbered ⅰ–ⅹ (Fig. 7(a)) to confirm them with the corresponding dislocations in the ECC images (Fig. 7(b)–(d)). We focused on the dislocations corresponding to ⅷ and ⅹ, observed in the BF-TEM image (Fig. 7(a)) and the ECC image from the front side of the sample (Fig. 7(b)) but not the ECC image from the back side (Fig. 7(c)). As a result, the dislocations corresponding to ⅷ and ⅹ were dislocations with endpoints at the surface and grain boundaries of the thin film. Furthermore, the dislocations corresponding to ⅶ and ⅸ were observed as whole dislocation lines in the BF-TEM image (Fig. 7(a)). In contrast, dislocation lines near the centers of ⅶ and ⅸ were not observed in ECC images because the dislocations penetrated through the front and back sides of the thin film, and the entire sample thickness could be observed in the BF-TEM image obtained at 300 kV. However, the entire sample thickness could not be observed because of the shallow information depth of SEM/ECCI at 30 kV; the dislocation contrast between BF-TEM and SEM/ECCI were obtained at the same diffraction vector in the same field of view, with good correspondence between them. The information depth of SEM/ECCI will be investigated in Section ‘Depth information measurement in SEM/ECCI by stereoscopic observation and quantitative analysis of dislocation density’. Thus, it was experimentally demonstrated that the SEM/ECCI method, where the channeling condition is strictly satisfied by controlling the incident electron beam direction, provides a contrast due to a single dislocation.

### Characteristic analysis of dislocation by SEM/ECCI

**Fig. 8.**
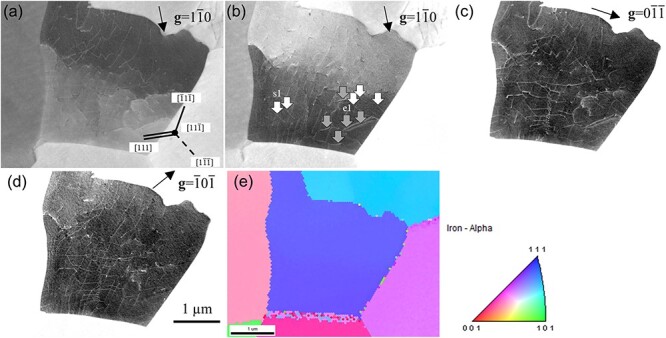
Characteristic analysis of dislocations using the **g⸱b **= 0 invisibility criterion; ECC images of ferrite grains at 30 kV with (a) ${\bf{g}} = 1\overline 1 0$, (b) tilted 0.8° about the axis parallel to $(1\overline 1 0)$ from (a), (c) ${\bf{g = }}0\overline 1 \overline 1 $ and (d) ${\bf{g}} = \overline 1 0\overline 1 $; (e) IPF map of grain observed in Fig. 8(a)–8(d).

**Fig. 9.**
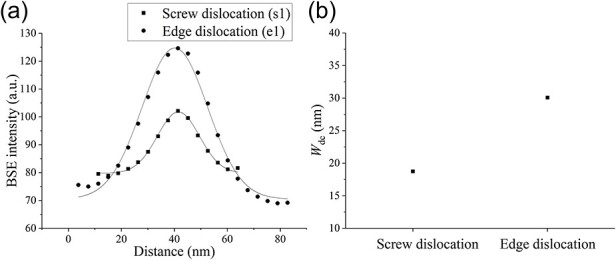
Comparison of dislocation width of screw and edge dislocations; (a) line profiles of BSE intensity along screw and edge dislocations (Fig. 8) named s1 and e1; (b) dislocation width $W_{\mathrm d\mathrm c}$ of screw and edge dislocations.

Figures 8(a)–(d) show ECC images of the same field using three different diffraction vectors **g**. Figure 8(e) shows the inverse pole figure (IPF) map of the observed grain, oriented approximately $[11\overline 1 ]$ perpendicular to the sample surface. The observed grain was slightly rotated about an axis parallel to $(1\overline 1 0)$ due to a low-angle grain boundary in the center. An image was taken with the top of the observed grains aligned in a channeling condition (Fig. 8(a)). Then, an image of the bottom of the observed grains was obtained by tilting the sample stage 0.8° around the axis parallel to $(1\overline 1 0)$ (Fig. 8(b)). For example, in the ECC image taken at ${\bf{g}} = \overline 1 0\overline 1 $, the dislocation contrast corresponding to the trace line of $[1\overline 1 \overline 1 ]$ disappeared (Fig. 8, white arrows). Assuming that the Burgers vectors **b** of the observed dislocation in the ferrite grain were *a*/2 <111>, the close-packed direction of body-centered cubic (BCC) (where *a* is lattice constant), then **b **= *a*/2 ± $[1\overline 1 \overline 1 ]$ and **b **= *a*/2 $[11\overline 1 ]$ satisfy the relationship **g⸱b **= 0 with $\mathbf g=\overline10\overline1$. Since ± $[1\overline 1 \overline 1 ]$ direction was perpendicular to the observed grain surface, **b** of the dislocation indicated by the opened arrows could be determined as **b **= *a*/2 ± $[1\overline 1 \overline 1 ]$. Furthermore, since the dislocation line vector **u** was parallel to the direction **b**, the dislocation could be determined as a screw dislocation with **b **= *a*/2 ± $[1\overline 1 \overline 1 ]$. Similarly, the dislocation characterization showed that most dislocations with lost dislocation contrast were screw dislocations. Additionally, edge dislocations with **b** and **u** orthogonal to each other were observed (gray arrows, Fig. 8). The BSE intensity profiles across the dislocation contrast of s1 and e1 (Fig. 8) are plotted in Fig. 9(a) to compare the dislocation contrast of screw and edge dislocations. $W_{\mathrm d\mathrm c}$ of the screw and edge dislocations (Fig. 8, white and gray arrows) were estimated, and the average $W_{\mathrm d\mathrm c}$ of screw and edge dislocations was plotted in Fig. 9(b). A difference in the width of the screw and edge dislocations existed; the edge dislocation line was wider than the screw dislocation line. In the calculation result of BF-TEM images in the dynamical theory [37,38], the width of a pure edge dislocation was about twice as wide as a pure screw dislocation, both lying parallel to the sample surface due to the difference in the strain field around the dislocations. The experimental results (Fig. 9) agreed with the calculation result of BF-TEM images. Therefore, the SEM/ECCI method can be proposed to determine the dislocation properties and observe the difference in the strain field of screw and edge dislocations, like the TEM method, because this method allows precise control of the electron beam incidence direction.

### Depth information measurement in SEM/ECCI by stereoscopic observation and quantitative analysis of dislocation density

**Fig. 10.**
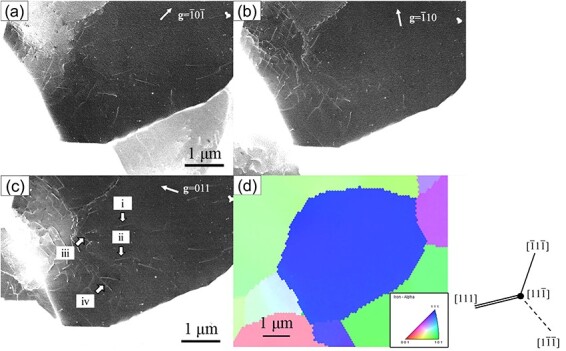
Characteristic analysis of dislocations using **g⸱b **= 0 invisibility criterion; ECC images of the ferrite grain at 30 kV with (a) ${\bf{g}} = \overline 1 0\overline 1 $, (b) ${\mathbf g{}=}\overline{{}1}10$ and (c) $\textstyle\mathbf g=011$, (d) inverse pole figure (IPF) map of grain observed in Fig. 10(a)–10(c).

**Fig. 11.**
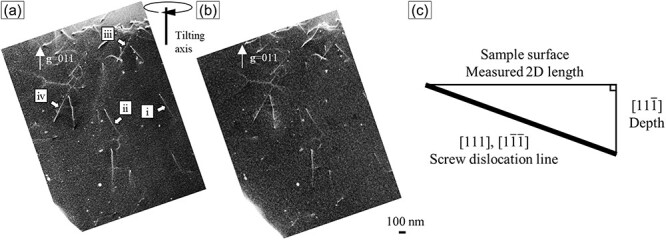
(a), (b) Pair of stereo ECC images at 30 kV tilted 12° with axis of **g **= ${\mathrm{011}}$; (c) geometric arrangement of the observed dislocations.

Understanding the depth of the image to quantify the dislocation density by SEM/ECCI is necessary. Regarding the depth information of SEM/ECCI, Wilkinson *et al*. [39] calculated that >90% of the BSE intensity was removed at 5*ξ*_g_ (*ξ*_g_ is the extinction distance) by applying the dynamical theory for SEM/ECCI proposed by Spencer *et al*. [6]. *ξ*_g_ is expressed by the following equation [2]:


(2) $$\xi_{\mathrm g}=\frac{\pi V_{\mathrm c}\mathrm c\mathrm o\mathrm s\theta_{\mathrm B}}{\lambda F}$$

where, *V*_c_ is the unit cell volume [m^−3^], and *λ* is the wavelength of the primary electron beam [m]. Gutierrez *et al*. [22] observed five oscillations in the stacking fault in high-Mn steel in the ECC image, where the frequency of the oscillations corresponded to *ξ*_g_. Nowadays, 5*ξ*_g_ is commonly used as the depth of ECC images [10,20,27]. However, a few examples have experimentally measured the depth information of dislocation contrast of steel in SEM/ECCI. If the geometrical arrangement of observation target is known, the depth information can be calculated from 2D length in a single ECC image. Therefore, the depth information of ECC images was investigated by stereoscopic observation, a technique where a pair of stereo images taken at different tilt angles are viewed in three dimensions using a stereo viewer or the like. This technique can accurately determine the direction of the dislocation line vector **u**. In this study, the depth information of the dislocation contrast in SEM/ECCI was determined using two-dimensional (2D) lengths of the dislocation contrast on the ECC image, and **u** obtained by stereoscopic observation. The characteristic analysis of the dislocation was carried out before the stereoscopic observation of the dislocation by SEM/ECCI. Figure 10 shows ECC images of the same field using three different diffraction vectors **g**. The observed grain was oriented approximately $[11\overline 1 ]$ perpendicular to the sample surface. Through the same argument as before, the dislocations numbered ⅰ and ⅱ in Fig. 10 can be determined to be dislocations with **b **= *a*/2 ± [111]; moreover, ⅲ and ⅳ can be identified as dislocations with **b **= *a*/2 ± $[1\overline 1 \overline 1 ]$. Figure 11(a) and (b) show a pair of stereo images taken at a 12° tilt around the tilt axis perpendicular to (${\mathrm{011}}$) crystal plane with **g **= ${\mathrm{011}}$ by SEM/ECCI. By stereo viewing of the stereo images, the dislocations corresponding to ⅰ and ⅱ could be identified as dislocations with **u **= ± [111]. Additionally, ⅲ and ⅳ could be identified to be dislocations with **u **= ± $[1\overline 1 \overline 1 ]$, not by trace matching. Therefore, the dislocations corresponding to ⅰ–ⅳ were almost pure screw dislocations. The 2D lengths of the dislocation lines corresponding to ⅰ to ⅳ were measured. Next, the depth information of the dislocation contrast in SEM/ECCI was estimated from the geometrical arrangement shown in Fig. 11(c). Table 1 lists the measured 2D lengths of the dislocation lines and the depth information. In this investigation, the depth information of the SEM/ECCI observation could be ∼170 ± 8 nm. In this case, for the ferrite grain of the iron BCC crystal structure with **g **= ${\mathrm{011}}$ at an accelerating voltage of 30 kV, 5*ξ*_g_ was ∼80 nm, about half of the measured depth information of this study. As discussed in Section ‘Influence of the angle of the primary electron beam direction in SEM/ECCI’, the sharpness of the dislocation contrast varied while deviating from the channeling conditions. Accordingly, SEM/ECCI, where the channeling condition is strictly satisfied by controlling the incident electron beam direction, can suggestively obtain dislocation contrast deeper than 5*ξ*_g_.

**Table 1.** Measured 2D length of dislocation lines in Fig. 7 and evaluated depth information

No.	ⅰ	ⅱ	ⅲ	ⅳ
2D length (nm)	481.7	492.9	481.3	469.8
Depth (nm)	170.3	174.3	170.2	166.1

**Fig. 12.**
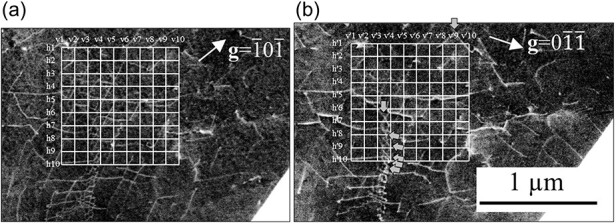
Grid for dislocation density measurement on ECC images at 30 kV with (a) **g **= $\overline 1 0\overline 1 $ and (b) **g **= $0\overline 1 \overline 1 $.

The dislocation density *ρ* [m^−2^] was measured by the grid intercept method [40] according to the equation


(3) $$\rho=\frac{n_1/l_1+n_2/l_2}t$$

where *n*_1_ and *n*_2_ are the number of intersections of the vertical and horizontal lines, *l*_1_ and *l*_2_ are the total length of the vertical and horizontal lines, and *t* is the visibility depth of the observation field. The measured and calculated depth values of 170 and 80 nm were used for *t*. Figure 12(a)–(b) shows the grid for measuring the dislocation density on ECC images, the same as Fig. 8(c)–(d). The grid consisted 10 vertical and 10 horizontal lines, 1 µm long. As discussed above, in the ECC image with **g **= $\overline 1 0\overline 1 $ the dislocations with **b **= *a*/2 ± $[1\overline 1 \overline 1 ]$ disappeared. Therefore, the ECC image with **g **= $0\overline 1 \overline 1 $ was used to measure the dislocation density. Table 2 lists the number of intersections between dislocation and grid lines. In the grid on the ECC image with **g **= $0\overline 1 \overline 1 $, the numbers of intersections counted only dislocations with **b **= *a*/2 ± $[1\overline 1 \overline 1 ]$, (gray arrows, Fig. 12(b)). The evaluated dislocation densities were 7.1 × 10^13^ and 1.5 × 1014 metre^−2^ at 170 and 80 nm. The dislocation density of well-annealed ferrite was ≤10^13^ metre^−2^; in this study, the SEM/ECCI measured depth value of 170 nm was considered a reasonable value.

**Table 2.** Number of intersections between dislocation and grid lines in Fig. 12

No.	h1	h2	h3	h4	h5	h6	h7	h8	h9	h10
Numbers of intersection	7	5	4	5	7	4	5	4	4	3
No.	h’1	h’2	h’3	h’4	h’5	h’6	h’7	h’8	h’9	h’10
Numbers of intersection	1	0	0	0	0	1	1	1	2	2
No.	v1	v2	v3	v4	v5	v6	v7	v8	v9	v10
Numbers of intersection	5	8	7	8	9	5	4	4	4	6
No.	v’1	v’2	v’3	v’4	v’5	v’6	v’7	v’8	v’9	v’10
Numbers of intersection	0	0	1	3	0	0	0	0	0	1

## Conclusion

SEM/ECCI was used to observe dislocations in steel materials under conditions where the intensity of the BSE was locally minimized and the incident primary electron beam direction on the crystal surface in SEM was precisely controlled. The direct comparison of the dislocation contrasts in the ECC images visualized under channeling conditions with the bright-field TEM images showed that the positions of all dislocation contrasts were in clear agreement. In the SEM/ECCI observation, the dislocation contrast varied depending on the incident electron beam direction of the crystal plane and accelerating voltages, and optimal conditions existed. When the diffraction condition **g** and the dislocation Burger vector **b** satisfied **g⸱b **= 0, the screw dislocation contrast in the ECC images disappeared. An edge dislocation line was wider than a screw dislocation line. Thus, the SEM/ECCI method could be used to characterize dislocation and evaluate the strain field around the dislocation, similar to the TEM method. The SEM/ECCI depth information, where the channeling conditions were strictly satisfied, could provide dislocation contrast deeper than 5*ξ*_g,_ typically used as the value of SEM/ECCI depth.

By precisely controlling the incidence primary electron beam direction on the crystal plane and taking ECC images, observing the same dislocation contrast as TEM observation is possible, even in steel materials requiring thin samples. In addition, the range of applications, such as using bulk samples for dislocation contrast imaging, will possibly expand and enable a wide range of observations.
