# Correction to: Observation and quantitative analysis of dislocations in steel using electron channeling contrast imaging method with precise control of electron beam incident direction

**DOI:** 10.1093/jmicro/dfae037

**Published:** 2024-08-24

This is a correction to: Takashige Mori, Takafumi Amino, Chie Yokoyama, Shunsuke Taniguchi, Takayuki Yonezawa, Akira Taniyama, Observation and quantitative analysis of dislocations in steel using electron channeling contrast imaging method with precise control of electron beam incident direction, *Microscopy*, Volume 73, Issue 4, August 2024, Pages 308–317, https://doi.org/10.1093/jmicro/dfad061

In the final paragraph of the ‘Experimental methods’ section, ‘110 μm aperture’ has been corrected to read ‘30 μm aperture’. In the title of Table 2, a reference to figure 8 has been corrected to read figure 12.

